# RETRACTED: Reddy et al. Mycotoxin Contamination of Beverages: Occurrence of Patulin in Apple Juice and Ochratoxin A in Coffee, Beer and Wine and Their Control Methods. *Toxins* 2010, *2*, 229–261

**DOI:** 10.3390/toxins2040934

**Published:** 2010-04-28

**Authors:** Florian Lang, Shu-Kun Lin

**Affiliations:** Editor-in-Chief, Toxins; Publisher, MDPI

It has been brought to our attention by a member of our Editorial Board that substantial portions of this review article [1] have been copied verbatim from earlier publications without credit. After comparing the present paper and the other sources we have determined that indeed this manuscript clearly violates our policy on originality of all material submitted for publication and the generally accepted ethics of scientific publication. Consequently, the Editorial Team and Publisher have determined that it should be retracted. We apologize for any inconvenience this may cause.
